# Associations of age with serum insulin, proinsulin and the proinsulin-to-insulin ratio: a cross-sectional study

**DOI:** 10.1186/1472-6823-10-21

**Published:** 2010-12-16

**Authors:** Bente Bryhni, Egil Arnesen, Trond G Jenssen

**Affiliations:** 1 Department of Cardiology, University Hospital of Tromsø, Tromsø, Norway; 2 Department of Clinical Medicine, University of Tromsø, Tromsø, Norway; 3 Institute of Community Medicine, University of Tromsø, Tromsø, Norway; 4 Department of Nephrology, Oslo University Hospital Rikshospitalet, Oslo, Norway

## Abstract

**Background:**

Insulin responses and insulin levels seem to decline with age. However, the question of beta cell impairment attributable to ageing has been sparsely addressed in population-based studies. Non-fasting insulin levels are determined by the ambient degree of insulin resistance together with the capacity of beta cells to compensate by insulin secretion to prevent hyperglycaemia. A raised proinsulin-to-insulin ratio (proinsulin/insulin) due to impaired processing of proinsulin is an early marker of beta cell dysfunction. We hypothesised that in a general population, signs of beta cell failure with advancing age manifest not only by decreases in random insulin, but also with a corresponding increase in its precursor proinsulin.

**Methods:**

In the Tromsø Study 1994-95 we measured insulin and proinsulin concentrations in random blood samples from 6212 persons without self-reported diabetes mellitus and plotted the levels as percentiles according to age. In regression analyses we assessed the relationships between age and insulin, proinsulin, and proinsulin/insulin, while adjusting for the concomitant measurements of glucose and other metabolic variables, and the time since the last meal.

**Results:**

Median insulin concentrations declined significantly with advancing age group in men, but not in women. Proinsulin levels and proinsulin/insulin increased across age groups in both genders. After adjustment, greater age was associated with lower log_10_(insulin) and higher log_10_(proinsulin) and log_10_(proinsulin/insulin) (p = 0.0001 for all).

**Conclusions:**

Negative associations of age with random insulin levels, together with positive associations of age with proinsulin and proinsulin/insulin, point towards a loss of beta cell function inherent in the ageing process.

## Background

The prevalence of abnormal glucose tolerance and diabetes mellitus increases with age [1-3]. For example, the age-specific prevalences of diabetes in Europe were less than 10% in subjects younger than 60 years, but between 10 and 20% in subjects 60-79 years of age [3]. A prerequisite for the development of impaired glucose tolerance (IGT) and type 2 diabetes is a decline in the capacity of the islet cells to secrete insulin. This deterioration of beta cell function prevents an adequate up-regulation of insulin secretion to compensate for insulin resistance [4].

Insulin release is reported to diminish with increasing age [5]. This decrease could involve a reduction in islet mass, but could also be caused by a functional impairment of the beta cells with ageing. A decline in post-challenge insulin levels with advancing age has been observed in population studies [6,7]. Although this finding could reflect beta cell failure in ageing, it might also be due to alterations in diet or gastric emptying, or even to an enhancement in insulin sensitivity in older age. Although proinsulin levels increase together with insulin concentrations in insulin resistance [8], a raised ratio of proinsulin to insulin, due to a disproportionate release of proinsulin from beta cells, is considered an early marker of islet dysfunction [4].

In 6212 men and women who had random measurements of insulin and proinsulin in a population-based study, and who did not report diabetes mellitus, we describe the percentiles of insulin and proinsulin levels and the proinsulin-to-insulin insulin ratio (proinsulin/insulin) according to gender and age. In the same persons we considered the influence of age on insulin, proinsulin, and the proinsulin/insulin ratio, after adjustment for the concomitant glucose concentrations, several covariates associated with insulin resistance, and the time since the last meal. Our hypothesis was that in a general population, signs of deterioration of beta cell function with advancing age is indicated not only by a decrease in random insulin levels, but also by a corresponding increase in proinsulin and proinsulin/insulin.

## Methods

### The Tromsø Study

The Tromsø Study was commenced in 1974 as a single centre prospective follow-up study of inhabitants in the municipality of Tromsø, with the primary objective to study cardiovascular risk factors. The fourth survey of the Tromsø Study [9] started in September 1994 and was completed in October 1995. The regional ethics committee approved the study and all subjects gave written informed consent. The study comprised two screening visits 4-12 weeks apart. All inhabitants older than 24 years were invited to the first visit (Phase 1) by a mailed letter, and 27,159 subjects (77% of the eligible population) attended. This examination included standardised measurements of height, weight, blood pressure, non-fasting serum lipids, serum calcium, γ-glutamyl transferase, haemoglobin and blood counts, and a 20-second ECG recording of lead I. All subjects aged 55-74 years and random 5-10% samples of the other age groups who attended the first examination were invited to a second visit (Phase 2) for a more extensive screening, and 7965 subjects attended (93% of those invited at Phase 1). This sample included women who had been invited separately for a study of osteoporosis [10] and persons who had participated in a family intervention trial [11].

### Study subjects

At the Phase 2 visit, 7407 persons (3177 men and 4230 women) provided blood samples that included insulin and proinsulin. One male and one female attendee with plasma insulin <6 pmol L^-1 ^were excluded. We excluded 957 women who had been invited separately to a study of osteoporosis [10]. Among these, 863 women aged 50-54 years were excluded as they were all examined between 08 and 09 AM, and the time from the last meal had not been recorded, and 94 (aged 25-34 years) were excluded because the handling of their blood samples was inadequate. Of the remaining, 111 men and 115 women were excluded due to self-reported diabetes mellitus or use of hypoglycaemic agents. The final sample included 6212 persons, of whom1116 had participated in a family intervention trial [11].

### Questionnaires

The first questionnaire (Additional file 1) was mailed with the invitation to participate in the study and was returned at the Phase 1 visit. A second questionnaire which differed in persons younger or older than 70 years (Additional files 2 and 3) was handed out at the Phase 1 visit, and returned by post. The first questionnaire inquired about whether the respondents had an established diagnosis of myocardial infarction, angina pectoris, or diabetes. Self-reported current smoking (cigarettes, cigars or pipe tobacco) and present antihypertensive medication were also inquired. Coffee consumption was registered as number of cups per day, alcohol intake as glasses per fortnight, and frequency of alcohol intake as occasions per month. A positive response to the question "are you a teetotaller" was taken as total abstinence from alcohol. Level of strenuous and light leisure-time physical activity was graded according to hours of activity resulting and not resulting in perspiration or breathlessness during an average week. The second questionnaire had questions about the use of hypoglycaemic agents.

### Examination at Phase 2

Height, weight, and waist and hip circumferences were measured with the subjects standing and wearing light clothing and no shoes, and body mass index (BMI) was calculated. Waist circumferences were measured at the umbilical line and hip girths at the widest circumferences, according to a written protocol. Blood pressure and heart rate (Dinamap Vital Signs Monitor 1846, Criticon Inc., Tampa, FL, USA) were recorded in a separate, quiet room before blood sampling. After the participants had been seated for 2 min, three measurements were made at 2-min intervals and the mean of the last two recordings used in the analyses. A nurse inquired again about diabetes, and use of medications in the preceding week, and the time from the last meal were recorded. Persons who reported that they took insulin or oral hypoglycaemic agents were also considered to have diabetes mellitus. Non-fasting venous blood samples were drawn between 0800 and 1600 h in seated persons. A brief venous stasis was released before sampling.

### Analytical methods

Serum insulin was measured by radioimmunoassay [12]. The insulin antibody had less than 0.2% cross-reactivity with proinsulin or its primary circulating split form, des (31,32)-proinsulin. Serum intact proinsulin was measured with a commercial kit (DAKO Diagnostics Ltd., Cambridgeshire, UK) using mouse monoclonal antibodies with no cross-reactivity to insulin and <0.1% cross-reactivity to split (32,33)-proinsulin. Plasma glucose was measured by a hexokinase method. Serum high-density lipoprotein (HDL)-cholesterol was measured after precipitation with manganese heparin and serum total cholesterol, triglycerides, uric acid, γ-glutamyl transferase, and creatinine by enzymatic colorimetric methods, all on a Hitachi 737 Automatic Analyzer (Boehringer Mannheim, Mannheim, Germany) with reagents from the same manufacturer. Haemoglobin, white blood cells, and platelets were analysed on a Coulter Counter (Beckman Coulter, Inc., Miami, FL, USA with reagents from Beckman Coulter Inc. (Fullerton, CA, USA). HbA_1c _was measured by an immuno-turbidimetric method on a Cobas Mira Plus Chemistry Analyzer (Roche Diagnostics, Basel, Switzerland) with reagents from the same company. The insulin and proinsulin samples were frozen at -70°C until analysis by the Metabolic Laboratory, Institute of Clinical Medicine, University of Tromsø in 1998 and 1999. All other samples were analysed by the Department of Medical Biochemistry, University Hospital of Tromsø.

### Statistical analyses

We constructed plots for the medians and 25 and 75 percentiles of insulin and proinsulin concentrations, as well as proinsulin/insulin according to gender and age group and computed the trends across age for the medians by regression analysis weighted for the inverse of the squared SEM in each age group. Age-adjusted percentages and means were calculated by the direct method. Age and covariates related to insulin resistance were considered in several multiple regression models and the best model identified. The following independent variables were examined: age, BMI, height, waist and hip circumferences, systolic and diastolic blood pressures, heart rate, total cholesterol, HDL-cholesterol, triglycerides, glucose, uric acid, creatinine, log_10_(γ-glutamyl transferase), white blood cell count, platelet count, calcium, fibrinogen, haemoglobin, coffee consumption, total abstinence from alcohol (yes = 1, no = 0), alcohol intake (times per month), current smoking (yes = 1, no = 0), use of antihypertensive medication (yes = 1, no = 0), use of beta blocker (yes = 1, no = 0), hours of light and strenuous physical activity per week and hours since the last meal. The final model was then employed with insulin, proinsulin and proinsulin/insulin as dependent variables, separately for men and women, and logarithmically transformed as the distributions were positively skewed. The regression coefficients (b) for the associations of the independent variables with the outcome variables were determined with the respective 95% confidence intervals (95% CI). We checked for interactions between age and the other independent variables by introducing interaction terms, and confirmed that the model assumptions were fulfilled. The data were analysed with the SAS 9.2 Statistical Package (SAS Institute Inc., Cary, NC, USA) and a two-sided p < 0.05 was considered statistically significant.

## Results

**Figure 1:**
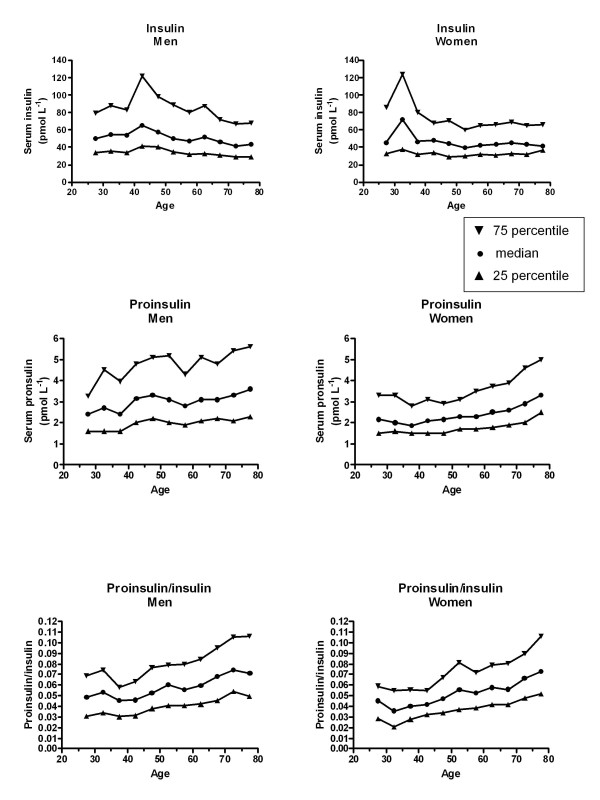
**Medians and 25 and 75 percentiles of serum insulin, proinsulin, and proinsulin/insulin by age**. Non-fasting persons without self-reported diabetes mellitus. The Tromsø Study 1994-95.

Table 1 summarises the mean age and age-adjusted characteristics of men and women. As illustrated in Figure 1 the median insulin levels decreased with advancing age group in men (p = 0.0104), but not in women (p = 0.5017). However, serum proinsulin (p = 0.0002 for women and p = 0.0066 for men) and proinsulin/insulin (p < 0.0001 for women and p = 0.0002 for men) rose across age strata in both genders. In multiple regression analysis, log_10_(insulin) was negatively whereas log_10_(proinsulin) and log_10_(proinsulin/insulin) positively associated with advancing age in both genders (Tables 2 and 3). Adjustments by HbA_1c _did not change the associations with age. Also, exclusion of either 38 men and 33 women with HbA_1c _at or above 6.5% (new criterion for the diagnosis of diabetes mellitus [13]) or 656 men and 726 women with HbA_1c _at or above 5.7% (the lower HbA_1c _level considered reasonable for identifying individuals with a high risk of future diabetes [13]) did not change the results. The significant associations with age also persisted after exclusion of persons who had participated in the family intervention trial [11] (data not shown). In men and women significant positive relationships were seen between smoking status and log_10_(proinsulin/insulin), but not log_10_(proinsulin). Elevated log_10_(proinsulin), but not log_10_(proinsulin/insulin) or log_10_(insulin), was associated with increased serum creatinine. Increased serum triglycerides were strongly related with elevated log_10_(insulin) and log_10_(proinsulin). Higher log_10_(insulin) and log_10_(proinsulin) were also associated with increased waist circumferences and log_10_(γ-glutamyl transferase) (Tables 2 and 3). Log_10_(insulin) and log_10_(proinsulin) were positively, but log_10_(proinsulin/insulin) inversely related with heart rate in both genders (Tables 2 and 3). The regression coefficients for the associations between log_10_(proinsulin) and the time since the last meal differed between men and women (p = 0.0167).

**Table 1:** Age and age-adjusted characteristics of study subjects (means ± SEM or %).

	Men		Women	
	**n**		**n**	

Age (years)	3065	59.6 ± 0.18	3147	60.6 ± 0.18

Serum insulin (pmol L^-1^)	3065	74.49 ± 1.64	3147	61.61 ± 1.26

Serum proinsulin (pmol L^-1^)	3053	4.18 ± 0.07	3141	3.22 ± 0.06

Plasma glucose (mmol L^-1^)	2993	4.80 ± 0.015	3083	4.74 ± 0.013

HbA_1c _(%)	2830	5.40 ± 0.009	2932	5.44 ± 0.010

BMI (kg m^-2^)	3061	25.96 ± 0.06	3141	25.82 ± 0.08

Waist circumference (cm)	3050	94.8 ± 0.17	3123	84.6 ± 0.19

Hip circumference (cm)	3050	103.1 ± 0.11	3123	103.1 ± 0.16

Systolic blood pressure (mmHg)	3062	141.04 ± 0.35	3130	138.52 ± 0.40

Heart rate (beats min^-1^)	3062	75.30 ± 0.24	3130	80.12 ± 0.24

Serum total cholesterol (mmol L^-1^)	3063	6.50 ± 0.022	3143	6.87 ± 0.024

Serum HDL-cholesterol (mmol L^-1^)	3058	1.39 ± 0.007	3141	1.66 ± 0.008

Serum triglycerides (mmol L^-1^)	3062	1.62 ± 0.018	3143	1.43 ± 0.014

Serum uric acid (μmol L^-1^)	3051	359.9 ± 1.63	3138	277.5 ± 1.30

Serum creatinine (μmol L^-1^)	3060	87.9 ± 0.39	3143	70.2 ± 0.23

Serum γ-glutamyl transferase (U L^-1^)	3063	32.7 ± 0.78	3144	24.3 ± 0.54

White blood cells (10^9 ^L^-1^)	2962	7.07 ± 0.04	3048	6.82 ± 0.03

Platelet count (10^9 ^L^-1^)	2960	239.2 ± 1.1	3049	256.5 ± 1.1

Plasma fibrinogen (g L^-1^)	3044	3.34 ± 0.02	3117	3.43 ± 0.01

Time since last meal (h)	3060	2.42 ± 0.04	3130	2.15 ± 0.03

**Questionnaire:**				

Myocardial infarction (%)	3059	9.0	3138	3.0

Angina pectoris (%)	3057	10.7	3142	6.8

Anti-hypertensive medication (%)	3065	13.2	3147	11.9

First degree relative with diabetes (%)	2436	21.7	2329	26.9

Current smoking (%)	3065	34.0	3147	31.5

Coffee consumption (cups day^-1^)	3064	6.06 ± 0.07	3145	4.90 ± 0.05

Teetotaller (%)	3063	13.2	3142	25.6

Alcohol intake (glasses month^-1^)	3057	3.18 ± 0.09	3132	1.62 ± 0.06

Light physical activity (h week^-1 ^)	3046	3.1	3142	3.0

Strenuous physical activity (h week^-1^)	3036	1.8	3110	1.5

BMI, body mass index; HDL, high-density lipoprotein

Non-fasting persons without self-reported diabetes mellitus. The Tromsø Study 1994-95

**Table 2:** Results of multiple regression analysis in men, with dependent variables log10(insulin), log10(proinsulin), and log10(proinsulin/insulin).

Independent variable	**Log**_**10**_**(insulin) (n = 2839**^**a**^**)** **Model R**^**2 **^**(adjusted) = 0.39**			**Log**_**10**_**(proinsulin) (n = 2840**^**a**^**)** **Model R**^**2 **^**(adjusted) = 0.33**			**Log**_**10**_**(proinsulin/insulin) (n = 2839**^**a**^**)** **Model R**^**2 **^**(adjusted) = 0.08**		
	**b**	**95% CI**	**p**	**b**	**95% CI**	**p**	**b**	**95% CI**	**p**

Age	-0.00281	-0.00381, -0.00181	<0.0001	0.00292	0.00194, 0.00390	<0.0001	0.00572	0.00470, 0.00674	<0.0001

BMI	0.01170	0.00625, 0.01715	<0.0001	0.00626	0.00089, 0.01163	0.0223	0.00544	-0.00009, 0.01097	0.0542

Waist circumference	0.00383	0.00171, 0.00595	0.0004	0.00440	0.00230, 0.00650	<0.0001	0.00057	-0.00159, 0.00273	0.6028

Systolic blood pressure	-0.00035	-0.00086, 0.00016	0.1719	-0.00034	-0.00085, 0.00017	0.1845	0.00002	-0.00049, 0.00053	0.9533

Heart rate	0.00307	0.00234, 0.00380	<0.0001	0.00165	0.00094, 0.00236	<0.0001	-0.00142	-0.00215, -0.00069	0.0001

Serum total cholesterol	-0.01523	-0.02332, -0.00714	0.0002	-0.01603	-0.02401, -0.00805	<0.0001	-0.00076	-0.00899, 0.00747	0.8565

Serum HDL-cholesterol	-0.07273	-0.09917, -0.04629	<0.0001	-0.06774	-0.09381, -0.04167	<0.0001	0.00499	-0.02192, 0.03190	0.7164

Serum triglycerides	0.05739	0.04426, 0.07052	<0.0001	0.06133	0.04837, 0.07429	<0.0001	0.00389	-0.00948, 0.01726	0.5680

Serum uric acid	0.00043	0.00029, 0.00057	<0.0001	0.00038	0.00024, 0.00052	<0.0001	-0.00005	-0.00019, 0.00009	0.5196

Serum creatinine	0.00033	-0.00010, 0.00076	0.1324	0.00115	0.00072, 0.00158	<0.0001	0.00082	0.00039, 0.00125	0.0003

Log_10_(γ-glutamyl transferase)	0.09858	0.05903, 0.13813	<0.0001	0.08575	0.04675, 0.12475	<0.0001	-0.01284	-0.05308, 0.02740	0.5317

Serum glucose	0.08659	0.07493, 0.09825	<0.0001	0.05416	0.04267, 0.06565	<0.0001	-0.03244	-0.04430, -0.02058	<0.0001

Time from last meal	-0.02926	-0.03314, -0.02538	<0.0001	-0.02782	-0.03164, -0.02400	<0.0001	0.00144	-0.00250, 0.00538	0.4755

Coffee consumption	-0.00429	-0.00682, -0.00176	0.0009	-0.00172	-0.00421, 0.00077	0.1772	0.00258	0.00001, 0.00515	0.0500

Current smoking	-0.04642	-0.06853, -0.02431	<0.0001	-0.00532	-0.02713, 0.01649	0.6326	0.04105	0.01855, 0.06355	0.0004

Non-fasting persons without self-reported diabetes mellitus. The Tromsø Study 1994-95.

^a^Used observations.; b, regression coefficient; 95% CI, 95% confidence interval; BMI, body mass index; HDL, high-density lipoprotein

**Table 3:** Results of multiple regression analysis in women with dependent variables log10(insulin), log10(proinsulin), and log10(proinsulin/insulin).

Independent variable	**Log**_**10**_**(insulin) (n = 2928**^**a**^**)** **Model R**^**2 **^**(adjusted) = 0.37**			**Log**_**10**_**(proinsulin) (n = 2928**^**a**^**)** **Model R**^**2 **^**(adjusted) = 0.31**			**Log**_**10**_**(proinsulin/insulin) (n = 2927**^**a**^**)** **Model R**^**2 **^**(adjusted) = 0.10**		
	**b**	**95% CI**	**p**	**b**	**95% CI**	**p**	**b**	**95% CI**	**p**

Age	-0.00404	-0.00496, -0.00312	<0.0001	0.00238	0.00146, 0.00330	<0.0001	0.00642	0.00544, 0.00740	<0.0001

BMI	0.00596	0.00177, 0.01015	0.0053	0.00266	-0.00151, 0.00683	0.2126	-0.00327	-0.00772, 0.00118	0.1494

Waist circumference	0.00333	0.00188, 0.00478	<0.0001	0.00284	0.00139, 0.00429	0.0001	-0.00046	-0.00201, 0.00109	0.5549

Systolic blood pressure	0.00029	-0.00012, 0.00070	0.1678	0.00022	-0.00019, 0.00063	0.3072	-0.00051	-0.00094, -0.00008	0.0229

Heart rate	0.00260	0.00197, 0.00323	<0.0001	0.00125	0.00062, 0.00188	0.0001	-0.00134	-0.00201, -0.00067	0.0001

Serum total cholesterol	-0.01216	-0.01904, -0.00528	0.0005	-0.01510	-0.02196, -0.00824	<0.0001	-0.00293	-0.01024, 0.00438	0.4321

Serum HDL-cholesterol	-0.02992	-0.05097, -0.00887	0.0054	-0.03564	-0.05665, -0.01463	0.0009	-0.00577	-0.02815, 0.01661	0.6131

Serum triglycerides	0.06114	0.04795, 0.07433	<0.0001	0.06283	0.04966, 0.07600	<0.0001	0.00168	-0.01233, 0.01569	0.8140

Serum uric acid	0.00049	0.00035, 0.00063	<0.0001	0.00056	0.00042, 0.00070	<0.0001	0.00007	-0.00009, 0.00023	0.3785

Serum creatinine	0.00047	-0.00016, 0.00110	0.1489	0.00151	0.00088, 0.00214	<0.0001	0.00105	0.00038, 0.00172	0.0023

Log_10_(γ-glutamyl transferase)	0.08676	0.05236, 0.12116	<0.0001	0.05826	0.02392, 0.09260	0.0009	-0.02875	-0.06530, 0.00780	0.1233

Serum glucose	0.09133	0.08028, 0.10238	<0.0001	0.05747	0.04644, 0.06850	<0.0001	-0.03390	-0.04564, -0.02216	<0.0001

Time from last meal	-0.02572	-0.02989, -0.02155	<0.0001	-0.02091	-0.02508, -0.01674	<0.0001	0.00479	0.00034, 0.00924	0.0346

Coffee consumption	-0.00322	-0.00626, -0.00018	0.0370	-0.00300	-0.00602, 0.00002	0.0522	0.00022	-0.00299, 0.00343	0.8952

Current smoking	-0.04636	-0.06637, -0.02635	<0.0001	-0.01223	-0.03220, 0.00774	0.2302	0.03402	0.01275, 0.05529	0.0017

Non-fasting persons without self-reported diabetes mellitus. The Tromsø Study 1994-95.

^a^Used observations; b, regression coefficient; 95% CI, 95% confidence interval; BMI, body mass index; HDL, high-density lipoprotein

## Discussion

In this cross-sectional study of persons without self-reported diabetes mellitus from the Tromsø Study we observed a decline in random casual concentrations of insulin across increasing age groups in men, but not in women. However, proinsulin levels and proinsulin-to-insulin ratios increased across age groups in both men and women. A decrease in insulin in both genders, together with a rise in proinsulin and in the proinsulin-to-insulin ratios with advancing age, emerged as significant after adjustment for the concurrent glucose levels, the time since the last meal, and covariates related to insulin sensitivity, which suggests that beta cell function deteriorates with age.

Cross-sectional data do not automatically allow inferences about changes in insulin and proinsulin over time or with age, as differences by cross-sectional age groups could reflect secular as well as age trends. Importantly, cross-sectional associations as in the present study, as opposed to longitudinal data, do not permit conclusions to be drawn about causality. However, although the data are not prospective, a large population-based sample of men and women, a high attendance rate, control of the numerous confounding factors, and record of the time since the last meal contribute to the validity of the results.

Whereas elevated fasting insulin is often considered as a marker of insulin resistance [14], stimulated insulin concentrations also convey information about the capacity of beta cells to secrete insulin relative to the level of insulin resistance [15]. Although the earliest secretory defect in the development of type 2 diabetes is a drop in the first-phase insulin release, a decreased capacity for maximal insulin release is seen prior to a reduction of basal insulin [16]. Due to this sequence of events, an age-related decline in insulin release might be more readily identified in non-fasting than in fasting samples. Whereas fasting insulin levels have been reported to increase [17] or not to change [18] with age in cross-sectional studies, a lower post-challenge insulin response was previously seen in elderly persons in the Baltimore Longitudinal Study of Aging [6] and in the Hoorn Study [7]. To our knowledge, the associations of proinsulin levels with age have not previously been addressed in population-based studies.

Hyperproinsulinaemia relative to insulin [4,19] indicates impaired processing of proinsulin, which is an early abnormality of beta cell function. Proinsulin concentrations are increased relative to insulin in both IGT [20] and type 2 diabetes [21]. Longitudinal data in older persons with IGT also indicate that the proinsulin-to-insulin ratio is a marker for progression to diabetes [22]. In persons with normal glucose tolerance there are conflicting results as to whether the proinsulin-to-insulin ratios increase with age [23-25]. Our results support the findings of Fritsche [25] and Shimizu and co-workers [24], who observed increases parallel with advancing age in both the proinsulin-to-insulin ratios after an oral glucose load [24,25] and the first phases of insulin release during hyperglycaemic clamps [25].

Although specific cut-offs for glucose concentrations have been settled for the diagnosis of diabetes mellitus [26], a deterioration of glucose control, and eventually the development of type 2 diabetes, is a process along a continuous scale. Notably, a proportion of our subjects probably had unidentified diabetes or IGT, and the increase in proinsulin/insulin with age observed in this study could conceivably just reflect the increase in individuals with abnormal glucose tolerance. However, a statement from the American Diabetes Association has recently affirmed that elevated HbA_1c _levels at or beyond 6.5% are sufficient to make a diagnosis of diabetes mellitus [13], and in addition, a HbA_1c _range of 5.7 to 6.4 is considered reasonable for identifying individuals with a high risk for future diabetes to whom the term prediabetes may be applied [13]. The omission of subjects with diabetes or prediabetes according to these definitions did not change the results.

In women, the median insulin levels did not decline across age groups, and a peak in insulin concentrations was observed in attendees aged 30-34 years. The menstrual cycle, pregnancy, and use of oral contraceptives are factors specific to women in this age group that may have influenced the insulin levels [27,28]. These issues, together with the onset of the menopause [27,29] and the possibility of hormone replacement therapy [29], are issues that complicate the consideration of the effect of age on insulin levels in women, and which could not be addressed in this study.

The amount of insulin released after the stimulus imposed by a meal varies according to its size and constitution, and the prevailing glucose concentrations [30]. Compared to gold standard methods to assess beta cell function, even indexes derived from an oral glucose tolerance test (OGTT) can only explain 27-64% of the variation in estimated beta cell function [15]. These variations are due to inter-individual differences in insulin responses from enteric hormones and neural responses to nutrient ingestion, gastrointestinal motility and gastric emptying [15,30]. Random insulin and proinsulin measurements, even if corrected for the time since the last meal, are certainly not comparable to those obtained by a standardised meal or an OGTT to assess beta cell function. Importantly however, non-fasting blood samples may be more clinically relevant than fasting specimens, as people remain in the non-fasting state a large part of the day [31]. Thus, insulin levels are involved in hypertriglyceridaemia after meals [32]. This postprandial hypertriglyceridaemia is linked with increased cardiovascular risk [31], and associated with abdominal obesity [32] and with increased liver fat and hepatic insulin resistance [33], as can be reflected by elevated γ-glutamyl transferase [34].

The reasons why older persons should fail to increase their insulin secretion as much as younger persons in the face of insulin resistance are incompletely understood. Potential mechanisms are reduced beta cell mass or limited beta cell function due to islet accumulation of amyloid, lipotoxicity, the actions of circulating adipocytokines, or a diminished effect of incretin hormones [4,5]. Alternative explanations for a negative association between age and serum insulin could be that older people preferentially eat meals that are smaller or have a composition to trigger less insulin release, or have slower rates of gastric emptying [35] and delayed nutrient absorption. Other causes could be selective mortality in elderly persons with elevated insulin due to impaired insulin action, or lower attendance rates in the older age groups because of illnesses associated with insulin resistance. However, the death rates of this population were not high enough to allow selective mortality to account for the results.

Because the fraction of total metabolic clearance accomplished by the kidneys is greater for proinsulin than for insulin [36], an age-related decline in renal function could conceivably have led to increased proinsulin concentrations relative to insulin, as we observed with advancing age. Advanced renal damage may also in itself affect glucose metabolism and both cause insulin resistance and impair insulin secretion [37]. However, as age, BMI, and serum creatinine were included in the regression equations and the analyses were performed separately for men and women, we did in effect adjust for the glomerular filtration rates [38]. Of note, however, since the half life of insulin and proinsulin are different [39], the kinetics of insulin differs from that of proinsulin in the postprandial state and direct comparisons of proinsulin/insulin ratios measured at different time points has not been validated.

Although smokers have been reported to be insulin resistant [40], decreased insulin concentrations in smokers have been described previously [7]. Not only did we observe negative associations between current smoking and insulin concentrations, but also positive associations between smoking and proinsulin/insulin. These observations are consistent with previous findings of diminished insulin secretion in smokers [41] and an observation in animal models that nicotine may have a detrimental effect directly on beta cell function [42].

Resting heart rate can be perceived as an integrated marker of haemodynamic and autonomic nervous system states, and is an independent predictor of cardiovascular disease [43]. Elevated heart rate may reflect a shift in autonomic balance toward enhanced sympathetic tone [43] and is associated with higher insulin [44] and proinsulin concentrations [45], as also observed in the present study. A novel finding of our study is a negative association in both genders between heart rate and proinsulin/insulin. This result is consistent with a previous report of a positive association between heart rate and the acute insulin response, as measured by a frequently sampled intravenous glucose tolerance test [45].

## Conclusions

The concurrent associations of lower insulin and higher proinsulin and proinsulin/insulin ratio with age in this cross-sectional study support a view that insulin release is blunted with advancing age, as marked by impaired processing of proinsulin to insulin. If taken together with previous reports [46,47], the data could also suggest that lifestyle interventions could offset beta cell dysfunction in predisposed elderly persons. However, this question will have to be investigated further.

## List of abbreviations

BMI: body mass index; CI: confidence interval; HDL: high-density lipoprotein; IGT: impaired glucose tolerance; OGTT: oral glucose tolerance test

## Competing interests

The authors declare that they have no competing interests.

## Authors' contributions

EA participated in the design, data management, statistical analyses, and drafting of the manuscript. BB participated in the design, performed statistical analyses, produced the figures, and drafted the manuscript. TGJ participated in the design and helped draft the manuscript. BB and TGJ read and approved the final manuscript.

## Pre-publication history

The pre-publication history for this paper can be accessed here:

http://www.biomedcentral.com/1472-6823/10/21/prepub

## Supplementary Material

Additional file 1 **First questionnaire in the Tromsø Study 1994-95**. English translation of the invitation with the first questionnaire used in the health survey in Tromsø 1994-95. Click here for file

Additional file 2 **Second questionnaire for subjects aged <70 years: The Tromsø Study 1994-95**. English translation of the second questionnaire used in the health survey in Tromsø 1994-95 for subjects younger than 70 years. Click here for file

Additional file 3 **Second questionnaire for subjects aged >70 years: The Tromsø Study 1994-95**. English translation of the second questionnaire used in the health survey in Tromsø 1994-95 for subjects 70 years or older. Click here for file
