# Supplementary material for: Associations of age with serum insulin, proinsulin and the proinsulin-to-insulin ratio: a cross-sectional study
Source: BMC Endocr Disord. 2010 Dec 16;10:21. doi: 10.1186/1472-6823-10-21 (PMC3020169; doi:10.1186/1472-6823-10-21)
Supplement: Additional file 1 — First questionnaire in the Tromsø Study 1994-95. English translation of the invitation with the first questionnaire used in the health survey in Tromsø 1994-95. [file 1472-6823-10-21-S1.PDF]

***English translation of invitation with the first questionnaire used in the health survey in Tromsø 1994/95***

Translation based on translations by Kevin McCafferty and Anne Clancy

# HEALTH SURVEY
# INVITATION

**"This is your chance"**

| | |
|---|---|
| Date of birth | Social security No. |
| Municipality | Electoral ward No. |

# Welcome to the Tromsø Health Survey!

The Health Survey is coming to Tromsø. This leaflet will tell you when and where. You will also find information about the survey in the enclosed brochure.

*We would like you to fill in the form overleaf and take it with you to the examination.*

The more people take part in the survey, the more valuable its results will be. We hope, therefore, that you will be able to come. Come along even if you feel healthy, if you are currently receiving medical treatment, or if you have had your cholesterol and blood pressure levels taken recently.

Yours sincerely,

Municipal Health Authorities

Faculty of Medicine - University of Tromsø

National Health Screening Service

**"This is a real opportunity — Take it!"**

**Your own health**

What is your current state of health?
*Tick one box only.*

| | |
|---|---|
| Poor | ☐ |
| Not so good | ☐ |
| Good | ☐ |
| Very good | ☐ |

Do you have, or have you ever had:

| | YES | NO | Age first time |
|---|---|---|---|
| Myocardial infarction | ☐ | ☐ | _____ years |
| Angina pectoris | ☐ | ☐ | _____ years |
| Stroke/ brain haemorrhage | ☐ | ☐ | _____ years |
| Asthma | ☐ | ☐ | _____ years |
| Diabetes | ☐ | ☐ | _____ years |

Do you take medicine for high blood pressure?

| | |
|---|---|
| At the moment | ☐ |
| Used to, but not any longer | ☐ |
| Never have | ☐ |

Have you during the last year suffered from pains and/or stiffness in muscles and joints that have lasted continuously for at least 3 months?
YES ☐ NO ☐

Have you in the last two weeks felt:

| | No | A little | A lot | Very much |
|---|---|---|---|---|
| Nervous or worried? | ☐ | ☐ | ☐ | ☐ |
| Anxious? | ☐ | ☐ | ☐ | ☐ |
| Secure and calm? | ☐ | ☐ | ☐ | ☐ |
| Irritable? | ☐ | ☐ | ☐ | ☐ |
| Happy and optimistic? | ☐ | ☐ | ☐ | ☐ |
| Down/depressed? | ☐ | ☐ | ☐ | ☐ |
| Lonely? | ☐ | ☐ | ☐ | ☐ |

**Smoking**

Did any of the adults at home smoke while you were growing up? YES ☐ NO ☐

Do you now, or have you previously, lived with daily smokers after your 20th birthday?
YES ☐ NO ☐

If "YES", for how many years in all? ____Years

How many hours a day do you normally spend in smoke-filled rooms? ________Hours
*Put 0 if you do not spend time in smoke-filled rooms.*

| Do you yourself smoke: | YES | NO |
|---|---|---|
| Cigarettes daily? | ☐ | ☐ |
| Cigars/cigarillos daily? | ☐ | ☐ |
| Pipe daily ? | ☐ | ☐ |

If you previously smoked daily, how long is it since you stopped? ______ Years

If you smoke daily at the moment, or have smoked before:

How many cigarettes do you smoke/did you smoke per day? ______ Cigarettes

How old were you when you began smoking daily? Age ______ Years

How many years in all have you smoked daily? ______ Years

**Exercise**

How has your physical activity in leisure time been during this last year? *Think of your weekly average for the year. Time spent going to work counts as leisure time.*

| | Hours pr. week | | | |
|---|---|---|---|---|
| | None | Less than 1 | 1-2 | 3 or more |
| Light activity (*not sweating or out of breath*) | ☐ | ☐ | ☐ | ☐ |
| Hard activity (*sweating/ out of breath*) | ☐ | ☐ | ☐ | ☐ |

**Coffee**

How many cups of coffee do you drink daily?
*Put 0 if you do not drink coffee daily.* Cups

Boiled coffee ☐☐☐
(i.e., grind boiled and allowed to draw)
Other coffee ☐☐☐

**Alcohol**

Are you a teetotaler? YES ☐ NO ☐

How many times a month do you normally drink alcohol? *Do not count low-alcohol beer.* ____ Times
*Put 0 if less than once a month.*

How many glasses of beer, wine or spirits do you normally drink in a fortnight? *Do not count low-alcohol beer. Put 0 if less than once a month.*

| Beer Glasses | Wine Glasses | Spirits Glasses |
|---|---|---|
| ☐ ☐ | ☐ ☐ | ☐ ☐ |

**Fat**

What kind of margarine or butter do you normally use on bread? *Tick one box only.*

- Don't use butter/margarine ☐
- Creamery butter ☐
- Hard margarine ☐
- Soft margarine ☐
- Butter/margarine blend ☐
- Light margarine ☐

**Education/work**

What is the highest level of education you have completed?

- 7-10 years primary/secondary school, modern secondary school, folk high school ☐
- Technical school, middle school, vocational.. school, 1-2 years' senior high school ☐
- A-levels/High school diploma, (3-4 years)☐

- College/university, less than 4 years ☐
- College/university, 4 or more years ☐

What is your current work situation?

- Paid work ☐
- Full-time housework ☐
- Education, military service ☐
- Unemployed, redundant ☐

How many hours of paid work do you have pr. week? _____ Hours

Do you receive any of the following benefits?

- Sickness benefit (sick leave) ☐
- Rehabilitation benefit ☐
- Disability pension ☐
- Old-age pension ☐
- Social welfare benefits ☐
- Unemployment benefit ☐

**Illness in the family**

Have one or more of your parents or siblings had a heart attack or had angina (heart cramp)?

| YES | NO | DON'T KNOW |
|---|---|---|
| ☐ | ☐ | ☐ |
