# Supplementary material for: Associations of age with serum insulin, proinsulin and the proinsulin-to-insulin ratio: a cross-sectional study
Source: BMC Endocr Disord. 2010 Dec 16;10:21. doi: 10.1186/1472-6823-10-21 (PMC3020169; doi:10.1186/1472-6823-10-21)
Supplement: Additional file 2 — Second questionnaire for subjects aged <70 years: The Tromsø Study 1994-95. English translation of the second questionnaire used in the health survey in Tromsø 1994-95 for subjects younger than 70 years. [file 1472-6823-10-21-S2.PDF]

English translation of the second questionnaire used in the health survey in Tromsø 1994/95 for subjects younger than 70 years.

Based on translations by K. McCafferty and A. Clancy

TROMSØ HEALTH SURVEY

The main aim of the Tromsø survey is to improve our knowledge of heart and circulatory conditions in order to aid prevention. The survey is also intended to improve our knowledge of cancer and other general conditions, such as allergies, muscle pains and nervous conditions. We would therefore like you to answer some questions about factors that may be relevant for your risk of getting these and other illnesses.

This form is part of the Health Survey, which has been approved by the Norwegian Data Inspectorate and the Regional Board of Research Ethics. The answers will only be used for research purposes and will be treated in strict confidence. The information you give us may later be stored along with information from other public health registers in accordance with the rules laid down by the Data Inspectorate and the Regional Board of Research Ethics.

If you are unsure about what to answer, tick the box that you feel fits best.

The completed form should be sent to us in the enclosed pre-paid envelope.

Thank you in advance for helping us.

Yours sincerely,

Faculty of Medicine — National Health
University of Tromsø — Screening Service

If you do not wish to answer the questionnaire, tick the box below and return the form. Then you will not receive reminders.

I do not wish to answer the questionnaire. ☐

Date for filling in this form: Day/Month/Year

CHILDHOOD/YOUTH

What Norwegian municipality did you live in at the age of 1 year? ___________________________

If you did not live in Norway, give country of residence instead of municipality.

How was your family's economic situation while you were growing up?

- Very good ☐
- Good ☐
- Difficult ☐
- Very difficult ☐

For how much of the first three years of your life
- did you live in a town/city? ____ Years
- did your family have a cat or dog in the home? ____ Years

For how much of the first 15 years of your life
- did you live in a town/city? ____ Years
- did your family have a cat or dog in the home? ____ Years

HOME

Who do you live with?

Tick once for each item and give the number of persons.

	YES	NO	Number
Spouse/partner	☐	☐	_____
Other persons over 18 years	☐	☐	_____
Persons under 18 years	☐	☐	_____

How many of the children go to day care/kindergarten/nursery school? ____

What type of home do you live in?

- Villa/ detached house ☐
- Farm ☐
- Flat /Apartment ☐
- Terraced /semi-detached house ☐
- Other ☐

How big is your home? _____ m2

Approximately what year was your home built? ______

	YES	NO
Has your home been insulated after 1970?	☐	☐
Do you live on the bottom floor/cellar level?	☐	☐
If "YES", is the floor laid on concrete?	☐	☐

What is the main source of heat in your home?

Electric heating	☐
Wood-burning stove	☐
Central heating system using:	
Paraffin	☐
Electricity	☐

	YES	NO
Do you have fitted carpets in the living-room?	☐	☐
Is there a cat in your home?	☐	☐
Is there a dog in your home?	☐	☐

WORK

If you are in paid or unpaid work, which statement describes your work best?

I am mainly seated while working (*e.g., at a desk/assembly work*)	☐
My work requires a lot of walking (*e.g., shop assistant, light industrial work, teaching*)	☐
My work entails a lot of walking and lifting (*e.g., postman/woman, nurse, building work*)	☐
I do heavy physical work (*e.g., forestry, heavy agricultural/construction work*)	☐

Do you have any influence on how your work is organised?

No, not at all	☐
To a small extent	☐
Yes, to a large extent	☐
Yes, I decide myself	☐

	YES	NO
Are you on call; do you work shifts or nights?	☐	☐

Do you do any of the following jobs (full- or part-time)?

Tick one box only for each item.	YES	NO
Driver	☐	☐
Farmer	☐	☐
Fisherman	☐	☐

YOUR OWN ILLNESSES

Have you ever had:

Tick one box only for each item. Give your age at the time. If you have had the condition several times, how old were you ***last time****?*

	YES	NO	AGE
Hip fracture	☐	☐	_____
Wrist/forearm fracture	☐	☐	_____
Whiplash	☐	☐	_____
Injury requiring hospital admission	☐	☐	_____
Stomach ulcer	☐	☐	_____
Duodenal ulcer	☐	☐	_____
An operation for stomach/ duodenal ulcer	☐	☐	_____
Throat/ neck operation	☐	☐	_____

Have you you ever had, or do you still have:

Tick one box only for each item.	YES	NO
Cancer	☐	☐
Epilepsy	☐	☐
Migraine	☐	☐
Chronic bronchitis	☐	☐
Psoriasis	☐	☐
Osteoporosis	☐	☐
Fibromyalgia/fibrositis/ chronic pain syndrome	☐	☐
Psychological problems for which you have sought help	☐	☐
Thyroid disease	☐	☐
Liver disease	☐	☐
Kidney stone	☐	☐
Appendectomy	☐	☐
**Allergy and hypersensitivity:**		
Atopic eczema (e.g., childhood eczema)	☐	☐
Hand eczema	☐	☐
Hay fever	☐	☐
Food allergy	☐	☐
Other hypersensitivity (not allergy)	☐	☐

How many times have you had a cold, influenza (flue), vomiting/diarrhoea, or similar in the last six months?

______ times

Have you had any of these in the last two weeks?

YES	NO
☐	☐

ILLNESS IN THE FAMILY

Tick the appropriate box for relatives that have, or have ever had the following illnesses: *Tick "None" if none of your relatives have had the condition.*

	Mother	Father	Brother	Sister	Child	None
Stroke or brain haemorrhage	☐	☐	☐	☐	☐	☐
Myocardial infarction before age 60	☐	☐	☐	☐	☐	☐
Cancer	☐	☐	☐	☐	☐	☐
Asthma	☐	☐	☐	☐	☐	☐
Stomach/ duodenal ulcer	☐	☐	☐	☐	☐	☐
Osteoporosis	☐	☐	☐	☐	☐	☐
Psychological problems	☐	☐	☐	☐	☐	☐
Allergy	☐	☐	☐	☐	☐	☐
Diabetes	☐	☐	☐	☐	☐	☐
-age when they got diabetes	___	___	___	___	___	___

SYMPTOMS

	YES	NO
Do you cough approximately every day of the year?	☐	☐
If "Yes": Is your cough productive ?	☐	☐
Have you had this kind of cough for as long as 3 months in each of the last two years?	☐	☐
Have you had periods of wheezing in your chest?	☐	☐
If "Yes", has this occurred: *Tick one box only for each item.*		
At night	☐	☐
In connection with respiratory infections	☐	☐
In connection with physical exertion	☐	☐
In connection with very cold weather	☐	☐
Have you noticed sudden changes in your pulse or heart rhythm in the last year?	☐	☐

How often do you suffer from sleeplessness?
- Never, or just a few times a year ☐
- 1-2 times a month ☐
- Approximately once a week ☐
- More than once a week ☐

If you suffer from periods of sleeplessness, what times of the year does it affect you most?
- No particular time of year ☐
- Especially during the dark winter months ☐
- Especially during the midnight sun period ☐
- Especially in spring and autumn ☐

Have you in the last twelve months suffered from sleeplessness to the extent that it has affected your ability to work? YES ☐ NO ☐

How often do you suffer from headaches?
- Seldom/Never ☐
- Once a month or more ☐
- Once a week or more ☐
- Every day ☐

Does the thought of getting a serious illness ever worry you?
- Not at all ☐
- Only a little ☐
- Some ☐
- Very much ☐

USE OF HEALTH SERVICES

How many visits have you made during the past year due to your own health or illness?*Tick **0** if you have **not** had such contact*

	Number of times the past year
To a general practitioner (GP)/ Emergency GP	_____
Psychologist or psychiatrist	_____
Other medical specialist (not at a hospital)	_____
Hospital out-patient clinic	_____
Hospital admission	_____
Medical officer at work	_____
Physiotherapist	_____
Chiropractor	_____
Acupuncturist	_____
Dentist	_____
Alternative medical practitioner (homoeopath, foot zone therapist, etc.)	_____
Healer, Faith healer, clairvoyant	_____

MEDICATION AND DIETARY SUPPLEMENTS

Have you for any length of time in the past year used any of the following medicines every day or almost daily? Indicate how many months you used them for.
*Write **0** for items you have **not** used.*

Medication:
- Painkillers _____ mths
- Sleeping pills _____ mths
- Tranquilizers _____ mths
- Antidepressants _____ mths
- Allergy drugs _____ mths
- Asthma drugs _____ mths

Dietary supplements
- Iron tablets _____ mths
- Calcium tablets or bonemeal _____ mths
- Vitamin D supplement _____ mths
- Other vitamin supplements _____ mths
- Cod liver oil or fish oil capsules _____ mths

Have you in the last 14 days used the following medicines or dietary supplements?
*Tick **one** box only for **each** item.*

Medicines	YES	NO
Painkillers	☐	☐
Antipyretic drugs (to reduce fever)	☐	☐
Migraine drugs	☐	☐
Eczema cream/ointment	☐	☐
Heart medicine (not blood pressure)	☐	☐
Lipid lowering drugs	☐	☐
Sleeping pills	☐	☐
Tranquilizers	☐	☐
Antidepressants	☐	☐
Other drugs for nervous conditions	☐	☐
Antacids	☐	☐
Gastric ulcer drugs	☐	☐
Insulin	☐	☐
Diabetes tablets	☐	☐
Thyroxin tablets (for metabolic disorder)	☐	☐
Cortisone tablets	☐	☐
Other medicine(s)	☐	☐

Dietary supplements	YES	NO
Iron tablets	☐	☐
Calcium tablets or bonemeal	☐	☐
Vitamin D supplement	☐	☐
Other vitamin supplements	☐	☐
Cod liver oil or fish oil capsules	☐	☐

FRIENDS

How many good friends do you have whom you can talk confidentially with and who give you help when you need it? ______ good friends
Do not count people you live with, but do include other relatives!

How many of these good friends do you have contact with at least once a month? ______

Do you feel you have enough good friends? YES ☐ NO ☐

How often do you normally take part in organised gatherings, e.g., sewing circles, sports clubs, political meetings, religious or other associations?

Never, or just a few times a year	☐
1-2 times a month	☐
Approximately once a week	☐
More than once a week	☐

DIET

If you use butter or margarine on your bread, how many slices does a small catering portion normally cover? By this, we mean the portion packs served on planes, in cafés, etc. (i.e., 10-12g)

A catering portion is enough for about ____ slices.

What kind of fat is normally used in **cooking** (not on the bread) in your home?

Creamery butter	☐
Hard margarine	☐
Soft margarine	☐
Butter/margarine blend	☐
Oils	☐

What kind of bread (bought or home-made) do you usually eat? *Tick one or two boxes!*
The bread I eat is most similar to

White bread	☐
Light textured brown bread	☐
Ordinary brown bread	☐
Coarse brown bread	☐
Crisp bread	☐

How much (in **number** of glasses, cups, potatoes or slices) do you usually eat or drink **daily** of the following foodstuffs? *Tick one box for **each** foodstuff.*

	0	Less than 1	1-2	3-4	5-6	More than 6
Full cream milk (fresh or soured) (glasses)	☐	☐	☐	☐	☐	☐
Semi-skimmed milk (low-fat) (fresh or soured) (glasses)	☐	☐	☐	☐	☐	☐
Skimmed milk (fresh or soured) (glasses)	☐	☐	☐	☐	☐	☐
Tea (cups)	☐	☐	☐	☐	☐	☐
Orange juice (glasses)	☐	☐	☐	☐	☐	☐
Potatoes	☐	☐	☐	☐	☐	☐
Slices of bread in total (incl. crispbread)	☐	☐	☐	☐	☐	☐

	0	Less than 1	1-2	3-4	5-6	More than 6
Slices of bread with fish (e.g., mackerel in tomato sauce	☐	☐	☐	☐	☐	☐
- lean meat (e.g., ham)	☐	☐	☐	☐	☐	☐
- fat meat (e.g., salami)	☐	☐	☐	☐	☐	☐
- cheese (e.g. Gouda/ Norvegia)	☐	☐	☐	☐	☐	☐
- brown cheese	☐	☐	☐	☐	☐	☐
- smoked cod caviar	☐	☐	☐	☐	☐	☐
- jam and other sweet spreads	☐	☐	☐	☐	☐	☐

How many **times per week** do you normally eat the following foodstuffs? *Tick a box for **all** foodstuffs listed.*

	Never	Less than 1	1	2-3	4-5	Roughly every day
Yoghurt	☐	☐	☐	☐	☐	☐
Boiled or fried egg	☐	☐	☐	☐	☐	☐
Breakfast cereal/ oat meal, etc.	☐	☐	☐	☐	☐	☐
For dinner						
- meat	☐	☐	☐	☐	☐	☐
- sausage/meatloaf/ meatballs	☐	☐	☐	☐	☐	☐
- fat fish (e.g., salmon/ redfish)	☐	☐	☐	☐	☐	☐
- lean fish (e.g., cod)						
- fishballs/fishpudding/ fishcakes	☐	☐	☐	☐	☐	☐
- vegetables	☐	☐	☐	☐	☐	☐
Mayonnaise, remoulade	☐	☐	☐	☐	☐	☐
Carrots	☐	☐	☐	☐	☐	☐
Cauliflower/cabbage/ broccoli	☐	☐	☐	☐	☐	☐
Apples/pears	☐	☐	☐	☐	☐	☐
Oranges, mandarines	☐	☐	☐	☐	☐	☐
Sweetened soft drinks	☐	☐	☐	☐	☐	☐
Sugarfree ("Light") soft drinks	☐	☐	☐	☐	☐	☐
Chocolate	☐	☐	☐	☐	☐	☐
Waffles, cakes, etc.	☐	☐	☐	☐	☐	☐

ALCOHOL

How often do you usually drink	beer?	wine?	spirits?
Never, or just a few times a year	☐	☐	☐
1-2 times a month	☐	☐	☐
Roughly once a week	☐	☐	☐
2-3 times a week	☐	☐	☐
Roughly every day	☐	☐	☐

Approximately how often in the last year have you drunk alcohol that equals at least 5 small bottles of beer, a bottle of wine, or 1/4 bottle of spirits?

Not in the last year	☐
Just a few times	☐
1-2 times a month	☐
1-2 times a week	☐
3 or more times a week	☐

For approximately how many years has your alcohol comsumption been as you described above? _____ years

WEIGHT REDUCTION

About how many times have you deliberately tried to lose weight? *Write **0** if you never have.*

- before age 20 _____ times
- after age 20 _____ times

If you have lost weight, about how many kilos have you ever lost at the most?

- before age 20 _____ times _____ kg
- after age 20 _____ times _____ kg

What weight would you be satisfied with (your "ideal weight")? _____ kg

URINARY INCONTINENCE

How often do you suffer from urinary incontinence?

Never ☐
Not more than once a month ☐
Two or more times a month ☐
Once a week or more ☐

Your comments:

Thank you for helping us! Remember to post the form today!

Tromsø Health Survey

TO BE ANSWERED BY WOMEN ONLY

MENSTRUATION

How old were you when you had your first menstruation? _____ years

If you no longer menstruate, how old were you when you stopped having menstruation? _____ years

Apart from pregnancy and after giving birth, have you ever stopped having menstruation for 6 months or more?
YES ☐ NO ☐

If "Yes", how many times? _____ times

If you still menstruate or are pregnant:
What date did your last menstruation begin?
day/month/year ___ / ___ / ___

Do you normally use painkillers to relieve period pains?
YES ☐ NO ☐

PREGNANCY

How many children have you given birth to? _____ children

Are you pregnant at the moment? YES ☐ NO ☐ Don't know ☐

During pregnancy, have you had high blood pressure and/or proteinuria? YES ☐ NO ☐

If "Yes", during which pregnancy?

	Pregnancy First	Later
High blood pressure	☐	☐
Proteinuria	☐	☐

If you have given birth, fill out for each child the year of birth and approximately how many months you breastfed the child.

Child:	Year of birth:	Number of months breastfed:
1	___________	______ months
2	___________	______ months
3	___________	______ months
4	___________	______ months
5	___________	______ months
6	___________	______ months

CONTRACEPTION AND OESTROGEN

Do you, or have you ever, used:	Now	Used to	Never:
Contraceptive pills (incl.minipill)	☐	☐	☐
A hormonal intrauterine device	☐	☐	☐
Oestrogen (tablets or patches)	☐	☐	☐
Oestrogen (cream or suppositories)	☐	☐	☐

If you use contraceptive pills, hormonal intrauterine device, or oestrogen, what brand do you currently use?

____

If you use, or have ever used, contraceptive pills:

Age when you began taking the pill? _____years

How many years in total have you taken the pill? _____years

If you have given birth, how many years did you take the pill before your first child? _____years

If you have stopped taking the pill:
Age when you stopped? _____years
