# Supplementary material for: Associations of age with serum insulin, proinsulin and the proinsulin-to-insulin ratio: a cross-sectional study
Source: BMC Endocr Disord. 2010 Dec 16;10:21. doi: 10.1186/1472-6823-10-21 (PMC3020169; doi:10.1186/1472-6823-10-21)
Supplement: Additional file 3 — Second questionnaire for subjects aged >70 years: The Tromsø Study 1994-95. English translation of the second questionnaire used in the health survey in Tromsø 1994-95 for subjects 70 years or older. [file 1472-6823-10-21-S3.PDF]

English translation of the second questionnaire used in the health survey in Tromsø 1994/95 for subjects 70 years or older.

Based on translations by Kevin McCafferty and Anne Clancy.

TROMSØ HEALTH SURVEY
for the over 70s

The main aim of the Tromsø survey is to improve our knowledge of heart and circulatory conditions in order to aid prevention. The survey is also intended to improve our knowledge of cancer and other general conditions, such as allergies, muscle pains and nervous conditions. The ultimate aim is to gain an overview of the general health of the elderly population. We would therefore like you to answer the questions below.

This form is part of the Health Survey, which has been approved by the Norwegian Data Inspectorate and the Regional Board of Research Ethics. The answers will only be used for research purposes and will be treated in strict confidence. The information you give us may later be stored along with information from other public health registers in accordance with the rules laid down by the Data Inspectorate and the Regional Board of Research Ethics.

If you are unsure about what to answer, tick the box that you feel fits best.

The completed form should be sent to us in the enclosed pre-paid envelope.

Thank you in advance for helping us.

Yours sincerely,

Faculty of Medicine
University of Tromsø

National Health
Screening Service

If you do not wish to answer the questionnaire, tick the box below and return the form. Then you will not receive reminders.

I do not wish to answer the questionnaire. ☐

Date for filling in this form: Day/Month/Year

CHILDHOOD/YOUTH

What Norwegian municipality did you live in at the age of 1 year?

If you did not live in Norway, give country instead of municipality.

How was your family's financial situation while you were growing up?

Very good	☐
Good	☐
Difficult	☐
Very difficult	☐

How old were your parents when they died?

Mother	______ years
Father	______ years

HOME

Who do you live with?
Tick one box for each item and give the number of persons.

	YES	NO	Number
Spouse/partner	☐	☐	
Other persons over 18 years	☐	☐	_______
Persons under 18 years	☐	☐	_______

What type of home do you live in?

Villa/detached house	☐
Farm	☐
Apartment/flat in block/terrace	☐
Terraced/semi-detached house	☐
Other	☐

How long have you lived in your present home? ____years

Is your home adapted to your needs? YES ☐ NO ☐

If "No", do you have problems with:

Space	☐
Variable temperature/too cold/too warm	☐
Stairs	☐
Toilet	☐
Bath/shower	☐
Maintenance	☐
Other (please specify)	☐

Would you like to move into a retirement home? YES ☐ NO ☐

PREVIOUS WORK AND FINANCIAL SITUATION

Which statement best describes the type of work you did for the last 5-10 years before you retired?

I was mainly seated while working (*e.g., desk/assembly work*)	☐
My work required a lot of walking (*e.g., shop assistant, housewife, teaching*)	☐
My work required a lot of walking and lifting (*e.g., postman, nurse, construction work*)	☐
I did heavy physical work (*e.g., forestry, heavy agricultural work, heavy construction work*)	☐

Did you do any of the following jobs (full- or part-time)?
Tick one box only for each item.

	YES	NO
Driver	☐	☐
Farmer	☐	☐
Fisherman	☐	☐

How old were you when you retired? ______ years

What kind of pension do you have?

Basic state pension	☐
Additional pension	☐

How is your current financial situation?

Very good	☐
Good	☐
Difficult	☐
Very difficult	☐

HEALTH AND ILLNESS

Has your state of health changed in the last year?

Yes, it has got worse	☐
No, unchanged	☐
Yes, it has got better	☐

How do you feel your health is now compared to others of your age?

Much worse	☐
A little worse	☐
About the same	☐
A little better	☐
Much better	☐

YOUR OWN ILLNESSES

Have you ever had:

Tick one box only for each item. Give your age at the time. If you have had the condition several times, how old were you ***last time****?*

	YES	NO	AGE
Hip fracture	☐	☐	_____
Wrist /forearm fracture	☐	☐	_____
Whiplash	☐	☐	_____
Injury requiring hospital admission	☐	☐	_____
Stomach ulcer	☐	☐	_____
Duodenal ulcer	☐	☐	_____
Stomach/duodenal ulcer operation	☐	☐	_____
Throat/neck surgery	☐	☐	_____

Have you ever had, or do you still have:

Tick one box only for each item.

	YES	NO
Cancer	☐	☐
Epilepsy	☐	☐
Migraine	☐	☐
Chronic bronchitis	☐	☐
Psoriasis	☐	☐
Osteoporosis	☐	☐
Fibromyalgia/fibrositis/ chronic pain syndrom	☐	☐
Psychological problems for which you have sought help	☐	☐
Thyroid disease	☐	☐
Liver disease	☐	☐
Thyroid disease	☐	☐
Liver disease	☐	☐
Recurrent urinary incontinence	☐	☐
Glaucoma	☐	☐
Cataract	☐	☐
Arthrosis (osteoarthritis)	☐	☐
Rheumatoid arthritis	☐	☐
Kidney stone	☐	☐
Appendectomy	☐	☐
Allergy and hypersensitivity		
Atopic eczema (e.g., childhood eczema)	☐	☐
Hand eczema	☐	☐
Hay fever	☐	☐
Food allergy	☐	☐
Other hypersensitivity (not allergy)	☐	☐

How many times have you had a cold, influenza (flue), diarrhea/vomiting, or similar in the last six months?

______ times

Have you had any of these in the last two weeks?

YES ☐ NO ☐

ILLNESS IN THE FAMILY

Tick off relatives who have, or have ever had, any of the following conditions:

Tick "None" for conditions which none of your relatives have had.

	Mother	Father	Brother	Sister	Child	None
Stroke or brain haemorrhage	☐	☐	☐	☐	☐	☐
Myocardial infarction before age 60	☐	☐	☐	☐	☐	☐
Cancer	☐	☐	☐	☐	☐	☐
Hypertension	☐	☐	☐	☐	☐	☐
Asthma	☐	☐	☐	☐	☐	☐
Osteoporosis	☐	☐	☐	☐	☐	☐
Arthrosis (osteoarthritis)	☐	☐	☐	☐	☐	☐
Psychological problems	☐	☐	☐	☐	☐	☐
Dementia	☐	☐	☐	☐	☐	☐
Diabetes	☐	☐	☐	☐	☐	☐
-age when they got diabetes	___	___	___	___	___	___

SYMPTOMS

	YES	NO
Do you cough daily for periods of the year?	☐	☐
If "Yes": Is your cough productive?	☐	☐
Have you had this kind of cough for as long as 3 months in each of the last two years?	☐	☐
Have you had periods of wheezing in your chest?	☐	☐

If "Yes", has this occurred:

Tick one box only for each item.

At night	☐	☐
In connection with respiratory infections	☐	☐
In connection with physical exertion	☐	☐
In connection with very cold weather	☐	☐
Have you noticed sudden changes in your pulse or heart rhythm in the last year?	☐	☐
Have you lost weight in the last year?	☐	☐

If "Yes":

How many kilograms? ______ kg

How often do you suffer from sleeplessness?

Never, or just a few times a year	☐
1-2 times a month	☐
Approximately once a week	☐
More than once a week	☐

If you suffer from periods of sleeplessness, what times of the year does it affect you most?

No particular time of year	☐
Especially during the 'dark winter months'	☐
Especially during the midnight sun period	☐
Especially in spring and autumn	☐

Do you usually take a nap during the day? YES ☐ NO ☐

Do you feel that you normally get enough sleep? YES ☐ NO ☐

Do you suffer from:	No	A little	A lot
Dizziness	☐	☐	☐
Poor memory	☐	☐	☐
Lack of energy	☐	☐	☐
Constipation	☐	☐	☐

Does the thought of getting a serious illness ever worry you?

Not at all	☐
Only a little	☐
Some	☐
Very much	☐

BODILY FUNCTIONS

Can you manage the following everyday activities on your own without help from others?

	Yes	With some help	No
Walking indoors on one level	☐	☐	☐
Walking up/down stairs	☐	☐	☐
Walking outdoors	☐	☐	☐
Walking approx. 500 metres	☐	☐	☐
Going to the toilet	☐	☐	☐
Washing yourself	☐	☐	☐
Taking a bath/shower	☐	☐	☐
Dressing and undressing	☐	☐	☐
Getting in and out of bed	☐	☐	☐
Eating meals	☐	☐	☐
Cooking	☐	☐	☐
Doing light housework (e.g., washing up)	☐	☐	☐
Doing heavier housework (e.g., cleaning floors)	☐	☐	☐
Going shopping	☐	☐	☐
Taking the bus	☐	☐	☐

	Yes	With difficulty	No
Can you hear normal speech (if necessary with a hearing aid)?	☐	☐	☐
Can you read (if necessary with glasses)?	☐	☐	☐

Are you dependent on any of the following aids?

	Yes	No
Walking stick	☐	☐
Crutches	☐	☐
Walking frame/Zimmer frame	☐	☐
Wheelchair	☐	☐
Hearing aid	☐	☐
Safety alarm device	☐	☐

USE OF HEALTH SERVICES

How many visits have you made during the past year due to your own health or illness:

*Tick **0** if you have **not** had such contact*

	Number of times the past year
To a general practitioner (GP)/ emergency GP	_____
Psychologist or psychiatrist	_____
Other medical specialist (not at a hospital)	_____
Hospital out-patient clinic	_____
Hospital admission	_____
Physiotherapist	_____
Chiropractor	_____
Acupuncturist	_____
Dentist	_____
Chiropodist	_____
Alternative medical practitioner (homoeopath, foot zone therapist, etc.)	_____
Healer, Faith healer, clairvoyant	_____

Do you have domestic help?	Yes	No
Private	☐	☐
Municipal	☐	☐
Do you receive services from the district nurse?	☐	☐

Are you pleased with the health care and home assistance services your municipality supplies?

	Yes	No	Don't know
Assigned family GP	☐	☐	☐
District nurse	☐	☐	☐
Home assistance	☐	☐	☐

Do you feel confident that you can receive the health care and home assistance you require if you need it?

Confident	☐
Not confident	☐
Very unsure	☐
Don't know	☐

MEDICATION AND DIETARY SUPPLEMENTS

Have you for any length of time in the past year used any of the following medicines every day or almost daily?
Indicate how many months you used them for.

*Write **0** for items you have **not** used.*

Medication:

Painkillers	______ mths
Sleeping pills	______ mths
Tranquillizers	______ mths
Antidepressants	______ mths
Allergy drugs	______ mths
Asthma drugs	______ mths
Heart medicine (not blood pressure)	______ mths
Insulin	______ mths
Diabetes tablets	______ mths
Thyroxin tablets (for metabolic disorder)	______ mths
Cortisone tablets	______ mths
Remedies for constipation	______ mths

Dietary supplements:

Iron tablets	______ mths
Vitamin D supplement	______ mths
Other vitamin supplements	______ mths
Calcium tablets or bonemeal	______ mths
Cod liver oil or fish oil capsules	______ mths

FAMILY AND FRIENDS

Do you have close relatives who can give you help and support when you need it? Yes ☐ No ☐

If "Yes", who can give you help?

Spouse/partner	☐
Children	☐
Others	☐

How many good friends do you have whom you can talk confidentially with and who give you help when you need it?

_______good friends

Do not count people you live with, but do include other relatives!

Do you feel you have enough good friends? Yes ☐ No ☐

Do you feel that you belong to a community or group of people who can depend on each other and who feel committed to each other (e.g., a political party, religious group, relatives, neighbours, work place, or organisation)?

- Strong sense of belonging ☐
- Some sense of belonging ☐
- Not sure ☐
- Little or no sense of belonging ☐

How often do you normally take part in organised gatherings, e.g., sewing circles, sports clubs, political meetings, religious or other associations?

- Never, or just a few times a year ☐
- 1-2 times a month ☐
- Approximately once a week ☐
- More than once a week ☐

DIET

How many meals a day do you normally eat (dinner and smaller meals)? _____ Number

How many times a week do you eat a hot dinner? _____ Number

What kind of bread (bought or home-made) do you usually eat? *Tick one or two boxes!*

The bread I eat is most similar to

- White bread ☐
- Light textured brown bread ☐
- Ordinary brown bread ☐
- Coarse brown bread ☐
- Crisp bread ☐

What kind of fat is normally used in **cooking** (not on the bread) in your home?

- Creamery butter ☐
- Hard margarine ☐
- Soft margarine ☐
- Butter/margarine blend ☐
- Oils ☐

How much (in **number** of glasses, cups, potatoes or slices) do you usually eat or drink **daily** of the following foodstuffs? *Tick one box for **each** foodstuff.*

	0	Less than 1	1-2	3-4	5-6	6-
Milk of all types (glasses)	☐	☐	☐	☐	☐	☐
Orange juice (glasses)	☐	☐	☐	☐	☐	☐
Potatoes	☐	☐	☐	☐	☐	☐
Slices of bread in total (incl. crispbread)	☐	☐	☐	☐	☐	☐
Slices of bread with fish (e.g., mackerel in tomato sauce)	☐	☐	☐	☐	☐	☐
- cheese (e.g., Norwegia)	☐	☐	☐	☐	☐	☐
- smoked cod caviar	☐	☐	☐	☐	☐	☐

How many **times per week** do you normally eat the following foodstuffs? *Tick a box for **all** foodstuffs listed.*

	Never	Less than 1	1	2-3	4-5	Roughly every day
Yoghurt	☐	☐	☐	☐	☐	☐
Boiled or fried egg	☐	☐	☐	☐	☐	☐
Breakfast cereal/ oat meal, etc.	☐	☐	☐	☐	☐	☐
For dinner						
- meat	☐	☐	☐	☐	☐	☐
- fat fish (e.g., salmon/ redfish)	☐	☐	☐	☐	☐	☐
- lean fish (e.g., cod)						
- vegetables (raw or cooked)	☐	☐	☐	☐	☐	☐
Carrots (raw or cooked)	☐	☐	☐	☐	☐	☐
Cauliflower/cabbage/broccoli	☐	☐	☐	☐	☐	☐
Apples/pears	☐	☐	☐	☐	☐	☐
Oranges, mandarines, etc.	☐	☐	☐	☐	☐	☐

WELL BEING

How content do you generally feel with growing old?

- Good ☐
- Quite good ☐
- Up and down ☐
- Bad ☐

What is your view of the future?

- Bright ☐
- Not too bad ☐
- Quite worried ☐
- Dark ☐

TO BE ANSWERED BY WOMEN ONLY

MENSTRUATION

How old were you when you had your first menstruation? _____ years

How old were you when you stopped having menstruations? _____ years

PREGNANCY

How many children have you given birth to? _____children

If you have given birth, fill out for each child the year of birth and approximately how many months you breastfed the child. If you have given birth to more than 6 children, note their birthyear and number of months you breastfed at the space provided below for comments.

Child:	Year of birth:	Number of months breastfed:
1	___________	_____________ months
2	___________	_____________ months
3	___________	_____________ months
4	___________	_____________ months
5	___________	_____________ months
6	___________	_____________ months

During pregnancy, have you had high blood pressure and/or proteinuria? Yes ☐ No ☐

If "Yes", during which pregnancy?

	Pregnancy First	Later
High blood pressure	☐	☐
Proteinuria	☐	☐

OESTROGEN

Do you, or have you ever used oestrogen:

	Now	Used to	Never
Tablets or patches	☐	☐	☐
Cream or suppositories	☐	☐	☐

If you use oestrogen, what brand do you currently use?

____

Your comments:

Thank you for helping us! Remember to post the form today! **Tromsø Health Survey**
